# *In Vitro* Interactions between Bacteria, Osteoblast-Like Cells and Macrophages in the Pathogenesis of Biomaterial-Associated Infections

**DOI:** 10.1371/journal.pone.0024827

**Published:** 2011-09-13

**Authors:** Guruprakash Subbiahdoss, Isabel C. Saldarriaga Fernández, Joana F. da Silva Domingues, Roel Kuijer, Henny C. van der Mei, Henk J. Busscher

**Affiliations:** Department of Biomedical Engineering, University Medical Center Groningen, Groningen and University of Groningen, Groningen, The Netherlands; University of California, Merced, United States of America

## Abstract

Biomaterial-associated infections constitute a major clinical problem that is difficult to treat and often necessitates implant replacement. Pathogens can be introduced on an implant surface during surgery and compete with host cells attempting to integrate the implant. The fate of a biomaterial implant depends on the outcome of this race for the surface. Here we studied the competition between different bacterial strains and human U2OS osteoblast-like cells (ATCC HTB-94) for a poly(methylmethacrylate) surface in the absence or presence of macrophages *in vitro* using a peri-operative contamination model. Bacteria were seeded on the surface at a shear rate of 11 1/s prior to adhesion of U2OS cells and macrophages. Next, bacteria, U2OS cells and macrophages were allowed to grow simultaneously under low shear conditions (0.14 1/s). The outcome of the competition between bacteria and U2OS cells for the surface critically depended on bacterial virulence. In absence of macrophages, highly virulent *Staphylococcus aureus* or *Pseudomonas aeruginosa* stimulated U2OS cell death within 18 h of simultaneous growth on a surface. Moreover, these strains also caused cell death despite phagocytosis of adhering bacteria in presence of murine macrophages. Thus U2OS cells are bound to loose the race for a biomaterial surface against *S. aureus* or *P. aeruginosa*, even in presence of macrophages. In contrast, low-virulent *Staphylococcus epidermidis* did not cause U2OS cell death even after 48 h, regardless of the absence or presence of macrophages. Clinically, *S. aureus* and *P. aeruginosa* are known to yield acute and severe biomaterial-associated infections in contrast to *S. epidermidis*, mostly known to cause more low-grade infection. Thus it can be concluded that the model described possesses features concurring with clinical observations and therewith has potential for further studies on the simultaneous competition for an implant surface between tissue cells and pathogenic bacteria in presence of immune system components.

## Introduction

Biomaterial-associated infection (BAI) is a serious problem in modern medicine. BAI is often difficult to treat, as the biofilm mode of growth protects infecting pathogenic microorganisms against both the host defense system and antibiotics [1]. In most cases, the final outcome is removal of the infected implant. Biomaterial implants can become contaminated by microorganisms in different ways. The best documented route is direct contamination of the implant surface during surgery (peri-operative contamination) or contamination during hospitalization (post-operative contamination). Whether or not microbial contamination eventually results in BAI, depends on the outcome of the so-called ‘race for the surface’ between successful tissue integration of the implant surface and biofilm formation [2]. If this race is won by tissue cells, then the implant surface is covered by a cellular layer and less vulnerable to biofilm formation. Alternatively, if the race is won by bacteria, the implant surface will become colonized by bacteria and tissue cell functions are hampered by bacterial virulence factors and excreted toxins [2], [3]. Since microorganisms are frequently introduced on an implant surface during surgery, microorganisms have a head start in this race for the surface. In the concept of the race for the surface, full coverage of an implant surface *in vivo* by a viable tissue cell layer, intact cell membrane and functional host defense mechanisms resists biofilm formation [4]. Especially in case of orthopedic and dental implants, establishment of a robust interface with fusion between biomaterial surface and bone tissue is essential, requiring adhesion, proliferation and differentiation of tissue cells for successful implantation.

*Staphylococcus epidermidis* and *Staphylococcus aureus* are the most frequently isolated pathogens from infected biomaterials implant surfaces [2], [5]. Additionally isolated organisms include *Escherichia coli* and *Pseudomonas aeruginosa* [2], [5]. Almost 50% of the infections associated with catheters, artificial joints and heart valves are caused by *S. epidermidis* [6], whereas *S. aureus* is detected in approximately 23% of infections associated with prosthetic joints [6]. *P. aeruginosa* is the causative organism of approximately 12% of hospital acquired urinary tract infections, 10% of bloodstream infections and 7% of hip joint infections [7].

Previously, we described an *in vitro* model to experimentally determine the influence of peri-operative bacterial contamination on the race for the surface, in which adhesion, spreading and growth of U2OS osteosarcoma cells on a biomaterial surface are compared in the absence or presence of adhering *S. epidermidis* [8]. The outcome of the competition between contaminating *S. epidermidis* ATCC 35983 and U2OS cells on glass appeared to be dependent on the number of bacteria adhering prior to U2OS cell seeding and the absence or presence of fluid flow. Cells lost the competition in the absence of flow conditions presumably due to accumulation of bacterial toxins, but were able to grow under flow due to the continuous supply of fresh medium to and removal of toxins from the interface on all commonly used biomaterial surfaces included in that study [9].

In a healthy host, the host immune system comes to the aid of tissue cells [10]. Macrophages are one of the most predominant immune cells that arrive within minutes to hours at an implant site and can remain at a biomaterial surface for several weeks to orchestrate the inflammatory process and foreign body reactions [10]. During infection, macrophages detect bacteria via cell surface receptors that bind to bacterial ligands and opsonines [11]–[13]. Subsequently, macrophages ingest pathogens and activate cellular functions such as proliferation, secretion of proteins and cytokines, and respiratory burst to destroy phagocytozed microorganisms and recruit other cells from the adaptive immune system [11]. However, it has been shown that the presence of a foreign body may impair the host immune system and consequently low numbers of adhering bacteria can already be sufficient to cause a BAI [14].

Bacterial virulence and alterations in the host defense including macrophage recruitment, are contributing factors to the pathogenesis of BAI [10], but hitherto have not been included in an experimental model to study the race for the surface. Therefore the aims of this study were to compare the influence of different bacterial strains of *S. epidermidis*, *S. aureus* and *P. aeruginosa* in a peri-operative contamination model on the outcome of the competition for a poly(methylmethacrylate) (PMMA) surface between bacteria and U2OS cells in the absence and presence of macrophages.

## Results

### Bacterial-U2OS cell interactions in absence of macrophages

**Figure 1:**
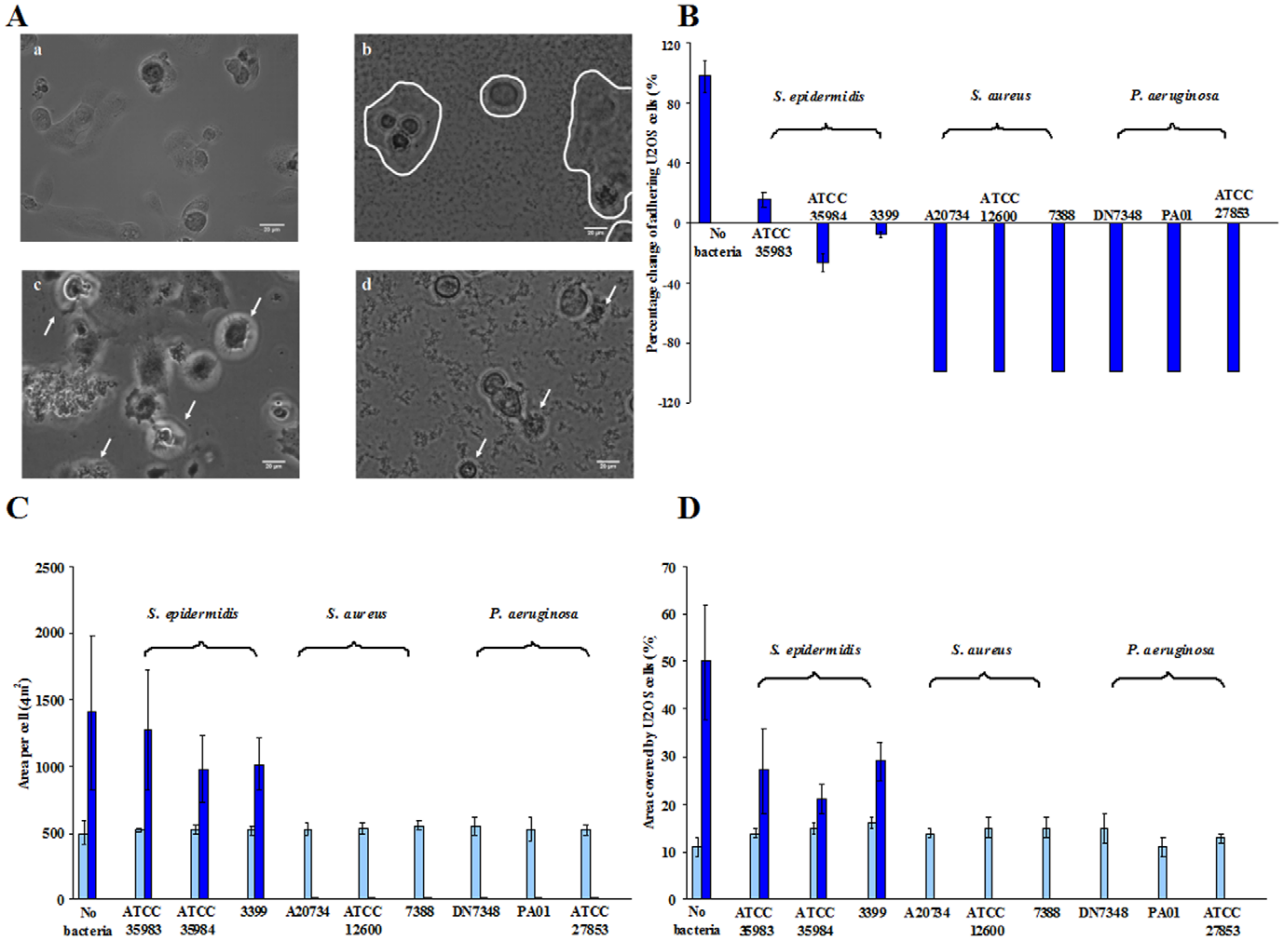
Simultaneous growth of bacteria and U2OS cells under flow. A) Phase contrast images of U2OS cells after 18 h of growth in the presence of adhering bacteria (a – control, b – *S. epidermidis* ATCC 35983, c – *S. aureus* ATCC 12600 and d – *P. aeruginosa* ATCC 27853) on PMMA surfaces. In Fig. 1A-b, U2OS cells are differentiated by a contour line from *S. epidermidis* biofilm. White arrows in Figs. 1A-c and 1A-d indicate U2OS cell death. The bar denotes 20 µm. B) Percentage change in the number of adhering U2OS cells after 48 h of growth with respect to their initial number immediately after seeding at 1.5 h on PMMA in the absence (no bacteria) and presence of adhering bacteria. Error bars represent the standard deviations over three replicates, with separately cultured bacteria and osteoblast-like cells. For *S. aureus* and *P. aeruginosa* the error bars are zero, since all percentage change in adhering numbers of U2OS cells were 100%. Cell number in the presence of bacteria is significantly different (p<0.01) from the cell number in the absence of bacteria. C) Average area per adhering U2OS cell immediately after seeding at 1.5 h (light blue) and after 48 h (dark blue) of growth on PMMA in the absence (no bacteria) and presence of adhering bacteria. Error bar represents the standard deviation over three replicates, with separately cultured bacteria and osteoblast-like cells. Average area by U2OS cells after 48 h in the presence of adhering bacteria (*S. aureus* and *P. aeruginosa*) is significantly different (p<0.05) from the absence of bacteria. D) Surface coverage by adhering U2OS cells immediately after seeding at 1.5 h (light blue) and 48 h (dark blue) of growth on PMMA in the absence (no bacteria) and presence of adhering bacteria. Error bar represents the standard deviation over three replicates, with separately cultured bacteria and osteoblast-like cells. Surface coverage by U2OS cells after 48 h in the presence of adhering bacteria are significantly different (p<0.05) from the absence of bacteria.

To compare the influence of different strains of *S. epidermidis*, *S. aureus* and *P. aeruginosa* in a peri-operative contamination model on the outcome of the competition for a PMMA surface between bacteria and U2OS cells, bacteria were allowed to adhere prior to U2OS cell adhesion and spreading. Subsequently, after 1.5 h of static adhesion of U2OS cells, simultaneous growth of bacteria and U2OS cells was allowed under flow at a shear rate of 0.14 1/s for a period of 48 h. Immediately after seeding, U2OS cell adhesion and spreading were observed using phase-contrast microscopy, both in the absence and presence of adhering bacteria on PMMA. At 1.5 h, the average number of adhering U2OS cells on the PMMA surface was 2.5×10^4^ cells/cm^2^ with an average area per cell of 500 µm^2^. The spreading of U2OS cells on the PMMA surface at 1.5 h was not significantly different in the absence or presence of adhering bacteria, regardless of whether *S. epidermidis*, *S. aureus* or *P. aeruginosa* strains were involved. After 18 h of growth, rounding up and detachment of U2OS cells, indicative of cell death, was observed on PMMA in the presence of adhering *S. aureus* and *P. aeruginosa* strains (Video S1), whereas no cell death was observed in the presence of adhering *S. epidermidis* strains (Figure 1A). Moreover, simultaneous growth of U2OS cells and *S. epidermidis* was observed for a period of 48 h (Video S2).

After 48 h of growth, TRITC-Phalloidin staining of the U2OS cells in combination with fluorescence microscopy was applied to quantitatively measure cell number and surface coverage. In Figure 1B, it can be seen that the number of adhering U2OS cells was significantly reduced (p<0.01) in the presence of *S. epidermidis* as compared to the control (no bacteria). The reduction in U2OS cell adhesion after 48 h was larger (p<0.01) in the presence of *S. epidermidis* 3399 and *S. epidermidis* ATCC 35984 compared to *S. epidermidis* ATCC 35983 (p<0.05). Adhering U2OS cells showed no significant difference in spreading per cell on PMMA in the presence of adhering *S. epidermidis* as compared to the control (Figure 1C).

**Figure 2:**
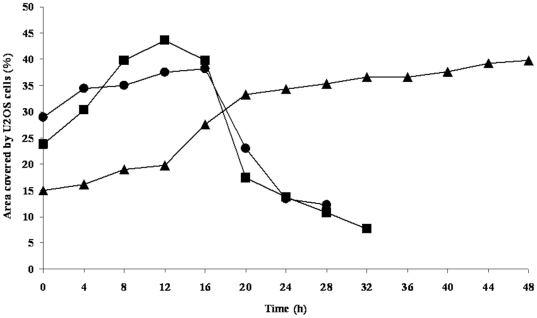
Surface coverage of U2OS cells as a function of time. An example of surface coverage by adhering U2OS cells as a function of time on PMMA in the presence of adhering bacteria ((▴) – *S. epidermidis* 3399, (▪) – *S. aureus* A20734, (•) – *P. aeruginosa* DN 7348).

In the concept of the race for the surface, the total surface coverage of the substratum by tissue cells is considered determinant for the fate of an implant. The coverage of the surface by U2OS cells at 1.5 h after seeding and after 48 h of growth are shown in Figure 1D. The surface coverage by adhering U2OS cells, 1.5 h after seeding varied between 11% and 16% regardless of the presence or absence of adhering bacteria. The coverage of the surface by U2OS cells was significantly increased after 48 h in the absence as well as in the presence of adhering *S. epidermidis*. In the presence of adhering *S. aureus* and *P. aeruginosa*, reductions in surface coverage by U2OS cells were observed after 18 h of growth (Figure 2), indicating U2OS cell death and no U2OS cells could be detected on the PMMA surface after 48 h.

### Bacterial-U2OS cell-macrophage interactions

To compare the influence of macrophages on the outcome of the competition for a PMMA surface between bacteria (*S. epidermidis*, *S. aureus* and *P. aeruginosa*) and U2OS cells, bacteria were allowed to adhere to the biomaterial surface prior to U2OS cell and macrophage adhesion. U2OS cells and macrophages were allowed to grow simultaneously for 24 h. Events are illustrated as follows:

#### Migration of macrophages towards bacteria and phagocytosis

**Figure 3:**
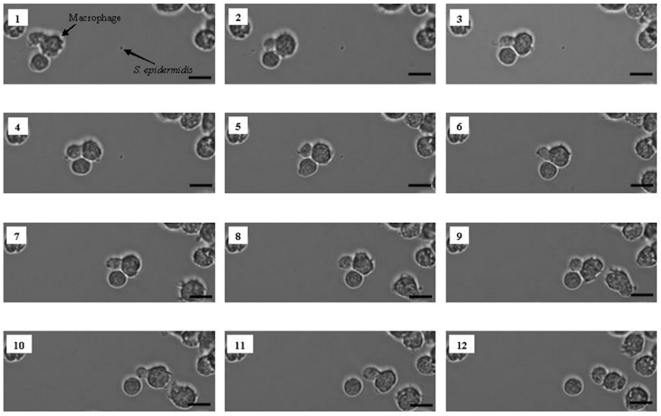
Macrophage migration towards *S. epidermidis* and phagocytosis under flow. Phase-contrast images of macrophage activity toward *S. epidermidis* ATCC 35983 on a PMMA surface in the presence of U2OS cells: macrophage migration towards *S. epidermidis* (images 1–5), bacterial clearance by phagocytosis (images 6–7) and further migration (images 8–12). The bar denotes 50 µm.

The number of bacteria adhering to the PMMA surface prior to seeding of U2OS cells and macrophages was set to 10^3^ bacteria/cm^2^. Subsequently, U2OS cells and macrophages were allowed to adhere to the surface and the simultaneous interactions between bacteria, macrophages and U2OS cells were observed by phase-contrast microscopy. As an example, Figure 3 shows macrophage migration in the presence of U2OS cells towards adhering *S. epidermidis* ATCC 35983 and subsequent phagocytosis. Macrophage migration towards bacteria and phagocytosis was similar on PMMA colonized by *S. epidermidis*, *S. aureus* and *P. aeruginosa* (data not shown).

#### Bacterial biofilm formation

**Figure 4:**
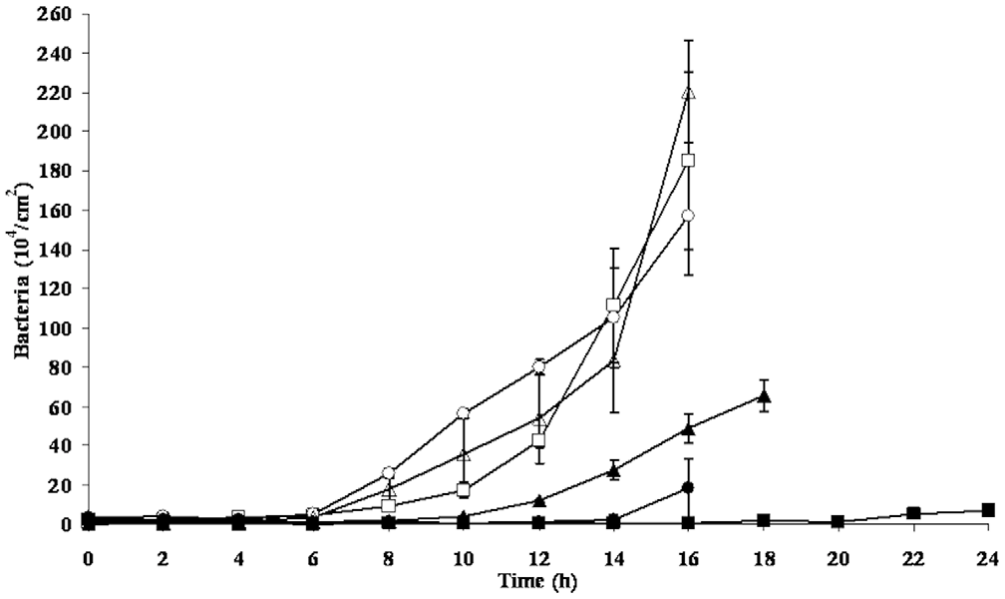
Restriction of bacterial biofilm growth by the presence of macrophages. The numbers of adhering bacteria on PMMA as a function of time during the simultaneous growth of bacteria and U2OS cells in the absence and presence of macrophages in a parallel plate flow chamber: *S. epidermidis* ATCC 35983 in the absence (□) and presence of macrophages (▪), *S. aureus* ATCC 12600 in the absence (○) and presence of macrophages (•), *P. aeruginosa* ATCC 27853 in the absence (▵) and presence of macrophages (▴).

Biofilm growth was assessed over time by determining the numbers of bacteria adhering to PMMA at different time points during the simultaneous growth of bacteria, U2OS cells and macrophages (Figure 4) for one strain of each of the three different bacterial species involved.

In the presence of macrophages, reductions in the numbers of adhering *S. epidermidis*, *S. aureus* and *P. aeruginosa* were observed as compared to absence of macrophages. This effect was seen up to 20 h of growth for *S. epidermidis* and up to 14 h and 10 h for *S. aureus* and *P. aeruginosa*, respectively. Thereafter, macrophage burst and release of ingested bacteria (predominantly alive) occurred.

#### U2OS cell adhesion and spreading

**Figure 5:**
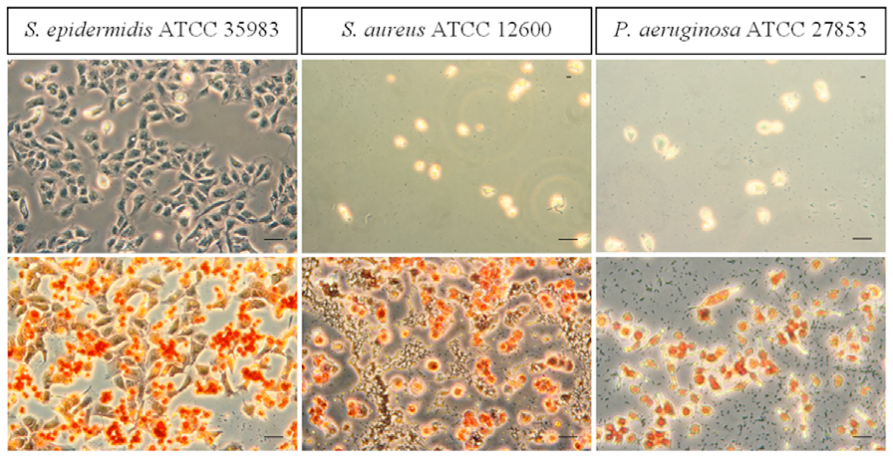
Adhesion and spreading of U2OS cells in the presence of macrophages. Phase-contrast images of adhering U2OS cells to PMMA after 24 h of simultaneous growth of bacteria (*S. epidermidis* ATCC 35983, *S. aureus* ATCC 12600 and *P. aeruginosa* ATCC 27853) and U2OS cells in the absence (upper images) and presence (lower images) of macrophages. Macrophages are orange-stained. The bar denotes 50 µm.

Immediately after seeding, U2OS cell adhesion and spreading on PMMA was observed, regardless of the presence or absence of macrophages. After 24 h of simultaneous growth U2OS cell death was observed in the presence of *S. aureus* and *P. aeruginosa* biofilms irrespective of the absence or presence of macrophages (Video S3). Alternatively, colonizing *S. epidermidis* did not significantly affect U2OS cells and their adhesion and spreading were similar both in the absence and presence of macrophages (see Figure 5).

## Discussion

This paper presents the first experimental *in vitro* model to study the influence of different pathogens on the outcome of the race between adhering bacteria and tissue cells for a biomaterial surface in the absence and presence of macrophages. In the model, bacteria were allowed to adhere prior to cell adhesion and spreading, which mimics the situation of peri-operative bacterial contamination of implant surfaces. The number of bacteria adhering on the PMMA surface prior to initiating U2OS cell adhesion and spreading was set to 10^3^ bacteria/cm^2^. Alternatively, we could have set the time allowed for bacterial contamination to occur as a constant, but this would have introduced the different abilities of the strains to adhere and grow into a biofilm into the study as an additional variable, which we wanted to avoid. In the past, it has been documented that during a surgical procedure of 1 h, the total number of bacteria carrying particles, falling on the wound is about 270 bacteria/cm^2^ [15]. These numbers are generally higher during periods of activity and when more people are present in the operation theatre [15]. More recent, through the use of modern, better ventilated operation theatres (20 changes of air per hour) and impermeable patient and personnel clothing, peri-operative bacterial contamination may be less [16]. However, many surgical procedures in which implants are introduced in the body last longer than 1 h. Therefore, the level of bacterial contamination chosen in our experiments is probably realistic. Due to these low numbers, peri-operatively introduced organisms, particularly when of low virulence, can survive on an implant surface for prolonged periods of time and later, during periods of host immune depression, proliferate and establish an infection with clinical symptoms [17].

The pathogenesis of BAI is complex and depends on factors such as bacterial virulence, physico-chemical properties of the biomaterial surface and alterations in the host defense [14]. Previously, in our model for the competition between *S. epidermidis* and U2OS osteoblast-like cells, all common biomaterial surfaces, including PMMA, allowed *S. epidermidis* ATCC 35983 biofilm formation with a negative impact on the coverage of the biomaterial surface by U2OS cells, although cells survived for at least 48 h [9]. Yet, PMMA showed better cell adhesion and spreading in the presence of adhering *S. epidermidis* ATCC 35983 than other commonly used biomaterials [9]. Our present study supports previous observations that U2OS cells are able to adhere, spread and grow in the presence of slime producing *S. epidermidis* strains, and extends these observations to the absence and presence of macrophages. Macrophages prolong the time that U2OS cells can survive an attack by adhering *S. aureus* and *P. aeruginosa*. However, it was observed that adhering *S. aureus* and *P. aeruginosa* cause death of all adhering U2OS cells and macrophages within 24 h. These observations are in line with clinical findings that BAI due to *S. aureus* and *P. aeruginosa* usually progresses much more aggressively than BAI caused by *S. epidermidis*. Buchholz and co-workers [18] showed that 54 of 64 patients (84%) with an infection by a low-virulent organism were free from infection 2 years later. Alternatively, when *S. aureus* was the causative organism, recurrent infection occurred in 28% of the patients, and almost 50% of the patients with a Gram-negative bacterial infection (*Pseudomonas*) experienced recurrent infection [18]. *S. aureus* appears more frequently in acute infections within 4 weeks after surgery of complicated total joint arthroplasty than does *S. epidermidis. S. epidermidis* is most commonly implicated in delayed septic loosening of total joint prostheses [19] or even in presumed aseptic loosening [20], indicating its low virulence with only minor clinical symptoms of infection [20]. This suggests a direct correlation between the clinical outcome and the causative bacterial strain, in line with our *in vitro* results. Overloading the system with a higher concentration of macrophages would eventually yield clearance of all adhering bacteria from the substratum surface in favor of U2OS cell adhesion and spreading, but considering the direct correlation existing between clinical outcome and the causative bacterial strain, we believe the current macrophage concentration to be most suitable for achieving the aim of this study, i.e. comparing the influence of different strains on the race for the surface.

The slime substance produced by *P. aeruginosa* along with other factors e.g. exotoxin A, exoenzyme S, elastase or alkaline protease accounts for the particular virulence of *Pseudomonas* in implant infections [7], [21]. *Pseudomonas* slime injected into mice produces liver and renal dysfunction and death within a short time, while no such effect is seen with *S. epidermidis* slime [21], [22]. In vascular grafts, pacemakers and orthopedic devices, *S. epidermidis* is mostly found in low-grade infections and a common cause of late infections. In patients with low-grade hip implant infections, only 5% of the patient had a temperature of 37.8°C or higher and only 20% had prolonged wound leakage [23]. The low virulence of *S. epidermidis* strains compared to *S. aureus* or *P. aeruginosa* is due to the lack of additional genes responsible for producing severely tissue damaging toxins [6], [7], [24]. In *S. epidermidis* infections, biofilm formation is considered the only virulence factor and therefore infections are usually sub-acute or chronic [25]–[27].

In general, immune cells migrate, engulf and kill invading microorganisms [28]–[30]. A previous study on the interaction between macrophages and colonizing *S. epidermidis*, showed that macrophage behavior is surface dependent [31]. Macrophage migration towards bacteria and phagocytosis was enhanced on highly hydrated, cross-linked poly(ethylene)-glycol (PEG) based polymer coatings compared to uncoated substrata due to the weak adhesion of macrophages and bacteria to the PEG coating [31]. On PMMA, we also see macrophages migrate towards adhering bacteria and engulf them. The degree of phagocytosis of adhering bacteria by macrophages differed depending on the virulence of the bacterial strain. In the presence of low-virulent *S. epidermidis*, bacterial biofilm growth was strongly reduced by the presence of macrophages up to 20 h compared to reductions lasting only 14 h and 10 h in the case of highly virulent *S. aureus* and *P. aeruginosa* biofilms, respectively. Furthermore, we observed that after a certain period of time, macrophages became exhausted and broke open which led to release of their bacterial content. Similarly, Kubica *et al.* [32] clearly showed that intracellular *S. aureus* can survive within human macrophages several days until a point where bacteria escaped the intracellular confinement, proliferated in the conditioned medium and killed the cells. Garzoni *et al.* [33] indicated that some coagulase-negative staphylococci could promote infection by intracellular colonization.

Several studies have demonstrated in line with the current results that immune cells may indeed lose their ability to kill bacteria in the presence of a biomaterial [30], [34], [35], [36]. Neutrophils exhibited decreased bactericidal activity and reduced superoxide production in the presence of extracellular slime producing *S. epidermidis* [37]–[41], while phagocytozed *S. aureus* suppressed the production of superoxide inside macrophages [36]. In a murine model, high numbers of *S. epidermidis* could not only persist within macrophages in peri-catheter tissue without showing any signs of inflammation [14], but were also able to proliferate. Macrophages in the periphery of a biomaterial *in vivo* showed deficient intracellular killing of pathogens, resulting in a compromised local host defense [14]. *In vivo*, bacteria may well survive inside the macrophages for prolonged periods of time. These bacteria will favor the development of BAI, especially when the physical condition of a patient disturbs the balance between bacteria and the host response [14].

The influence of macrophages described here on the competition between bacteria and tissue cells to colonize and integrate a biomaterial surface is novel. It is demonstrated that despite the presence of macrophages, U2OS cells loose the race for the surface in the presence of highly virulent *S. aureus* or *P. aeruginosa*, while cells can survive at least 48 h in the presence of *S. epidermidis*, regardless of the absence or presence of macrophages.

These results clearly bear features concurrent with clinical observations and therewith the model described, including the bacterial and macrophage densities used and the use of an immortal human cell line derived from osteosarcoma cells, has great potential to study the simultaneous competition for an implant surface between tissue cells and bacteria in the presence of immune system components.

## Materials and Methods

### Biomaterial

Poly(methyl methacrylate) (PMMA) (Vink Kunststoffen, Didam, The Netherlands) was used as a substratum. Samples were rinsed thoroughly with ethanol (Merck, Darmstadt, Germany) and washed with sterile ultrapure water before use to yield a water contact angle of 73±3 degrees [9].

### Osteoblast-like cell culturing and harvesting

U2OS osteoblast-like cells, an immortal human cell line derived from osteosarcoma cells, were chosen for this study because of their ease of growth, although we realize that cancer cell lines may not represent all aspects of *in vivo* cell behavior and possibly differ in integrin expression, with an impact on their adhesion to fibronectin-coated biomaterials. U2OS osteosarcoma cells (ATCC number: HTB-96; obtained from LGC standards, Wesel, Germany) [8] are routinely cultured in Dulbecco's modified Eagles Medium (DMEM)-low glucose supplemented with 10% fetal calf serum (FBS), 0.2 mM ascorbic acid-2-phosphate (AA2P), denoted in the paper as “DMEM+FBS”. Cells were maintained at 37°C in a humidified atmosphere with 5% CO_2_, and were passaged at 70–90% confluency using trypsin/ethylenediaminetetraacetic acid. The harvested cells were counted using a Bürker-Türk haemocytometer and subsequently diluted to a concentration of 6×10^5^ cells/ml.

### Macrophages culturing and harvesting

J774A.1 murine macrophages (ATCC number: TIB-67; obtained from LGC standards, Wesel, Germany) were routinely cultured in DMEM-high glucose supplemented with 10% FBS and denoted in the paper as “optimal medium”. Macrophages were maintained at 37°C in a humidified atmosphere with 5% CO_2_, and passaged at 70–80% confluency by scraping. The harvested cells were counted using a Bürker-Türk haemocytometer and subsequently diluted to a concentration of 12×10^5^ cells/ml. The macrophage J774A.1 was chosen because this cell line does not grow in suspension and adheres but hardly spreads on a substratum surface.

### Bacterial growth conditions and harvesting

The bacterial strains used in this study were *S. epidermidis* ATCC 35983, *S. epidermidis* ATCC 35984, *S. epidermidis* 3399 (isolated from skin), *S. aureus* ATCC 12600, *S. aureus* A20734 (isolated from an infected abdominal mesh), *S. aureus* 7388 (isolated from an infected joint prosthesis), *P. aeruginosa* DN7348 (isolated from an infected joint prosthesis), *P. aeruginosa* PA01 (clinical isolate from burn wound), *P. aeruginosa* ATCC 27853. All *S. epidermidis* strains used were slime producing, as indicated by a Congo-red agar assay (see Table S1). First, a strain was streaked on a blood agar plate from a frozen stock and grown overnight at 37°C. The plate was then kept at 4°C. For each experiment, a colony was inoculated in 10 ml of tryptone soya broth (TSB; OXOID, Basingstoke, England) and cultured for 24 h. This culture was used to inoculate a second culture, which was grown for 17 h prior to harvesting. Bacteria were harvested by centrifugation at 5000× g for 5 min at 10°C and washed twice with sterile ultrapure water. Subsequently, the harvested bacteria were sonicated on ice (3×10 s) in sterile potassium phosphate buffer (PBS, 10 mM potassium phosphate, 0.15 M NaCl, pH 7.0) in order to break bacterial aggregates. This suspension was further diluted in sterile PBS to a concentration of 3×10^6^ bacteria/ml. Prior to the experiments, growth and biofilm formation of all bacterial strains in modified culture medium (98% DMEM+FBS and 2% TSB [8]) was confirmed by culturing bacteria in this medium for 48 h.

### Ethics statement

Cell lines used in this study were obtained from LGC standards, Wesel, Germany.

### Competitive assay for U2OS cell growth and biofilm formation

The competitive assay was performed on the PMMA bottom plate of a parallel plate flow chamber (175×17×0.75 mm^3^), as described in detail before [8]. The flow chamber was equipped with heating elements and kept at 37°C throughout the experiments. Bacterial and U2OS deposition were observed with a CCD camera (Basler AG, Germany) mounted on a phase-contrast microscope Olympus BH-2 (Olympus, Germany) with a 40× objective for bacteria and 10× objective for U2OS cells.

Prior to each experiment, all tubes and the flow chamber were filled with sterile PBS, taking care to remove all air bubbles from the system. Once the system was filled, and before the addition of the bacterial suspension, PBS was allowed to flow through the system at a shear rate of 11 1/s. Then, a bacterial suspension in PBS was perfused through the chamber at the same shear rate and phase-contrast images were obtained. As soon as the desired density of adhering bacteria (10^3^ bacteria/cm^2^), was reached, flow was switched to sterile PBS to remove the bacterial suspension from the tubes and chamber. Subsequently, a U2OS cell suspension (6×10^5^ cells/ml) in modified culture medium was allowed to enter the flow chamber. Once the entire volume of buffer inside the chamber was replaced by the cell suspension, flow was stopped for 1.5 h in order to allow U2OS cells to adhere and spread on the substratum surface. Subsequently, phase contrast images (nine images, 900×700 µm each) were taken and the number of adhering cells per unit area as well as the area per spread cell were determined using Scion image software. Finally, modified culture medium supplemented with 2% HEPES was perfused through the system at a shear rate of 0.14 1/s for 48 h and phase-contrast images were obtained every 30 min. Biofilm growth was assessed in real-time by determining the numbers of adhering bacteria per unit area using proprietary software based on the Matlab Image processing Toolkit (The MathWorks, MA, USA).

### Fluorescence staining and determination of material surface coverage with U2OS cells

After 48 h of flow, the surfaces were prepared for fluorescence staining to assess cell number and surface coverage. Phase contrast images could not be used for quantification because the images were too crowded after 48 h and the spread area per cell was difficult to determine. For fixation, surfaces with adhering bacteria and U2OS cells were placed in a Petri dish with 30 ml of 3.7% formaldehyde in cytoskeleton stabilization buffer (CS; 0.1 M Pipes, 1 mM EGTA, 4% (w/v) polyethylene glycol 8000, pH 6.9). After 5 min, the fixation solution was replaced by 30 ml fresh CS for another 5 min. Subsequently, U2OS cells were incubated in 0.5% Triton X-100 for 3 min, rinsed with PBS and stained for 30 min with 5 ml PBS containing 49 µl DAPI and 2 µg/ml of TRITC-phalloidin and the U2OS cells on the surfaces were washed four times in PBS. DAPI will stain the nucleus blue, while TRITC-phalloidin stains the actin cytoskeleton in red. Images (nine images on different locations, 900×700 µm each) were taken with a confocal laser scanning microscopy (Leica DMRXE with confocal TCS SP2 unit). The images were analyzed using Scion image software and the number of adhering U2OS cells per unit area and the average area per spread cell were determined. The total coverage of the substratum surface by U2OS cells was calculated from the number of cells and the spread area.

### Competitive assay for U2OS cell growth and biofilm formation in the presence of macrophages

The competition between bacteria and U2OS cells for the colonization of PMMA in the presence of macrophages was assessed on the PMMA for one strain of each of the three different bacterial species involved, roughly according to the above procedure with some minor modification.

As soon as the desired density of adhering bacteria (10^3^ bacteria/cm^2^), was reached and flow was switched to sterile PBS, a cell suspension consisting of U2OS cells (6×10^5^ cells/ml) and J774A.1 macrophages (12×10^5^ cells/ml) in optimum medium was added to the flow chamber. Once the entire volume of buffer inside the chamber was replaced by the cell suspension, flow was stopped for 1.5 h in order to allow U2OS cells and macrophages to adhere and spread on the substratum surface. Ultimately, optimal medium supplemented with 2% HEPES was perfused through the system at a shear rate of 0.14 1/s for 24 h and phase-contrast images were obtained continuously at 2 min intervals. Biofilm growth was assessed in real-time by determining the numbers of adhering bacteria per unit area.

At the end of the assay, surfaces were prepared for qualitative analysis to assess U2OS cell and macrophage shape and spreading. Cells adhering to PMMA were fixed with citrated-acetone-formaldehyde fixative solution for 30 s and stained with an alkaline-dye mixture (Sigma-Aldrich, Germany) (Naphtol AS-BI phosphate, sodium nitrite, fast blue BB base) for 15 min. Samples were subsequently rinsed with demineralized water and counterstained for 2 min with neutral red solution. Then the samples were rinsed once again with demineralized water, allowed to dry and phase-contrast images were taken on different places of the sample. Differentiated U2OS osteosarcoma cells stained purple/blue (alkaline phosphatase-positive) and macrophages were orange stained.

### Statistics

Data are presented as a mean with standard deviation. Statistical ANOVA analysis was performed followed by a Tukey's HSD post-hoc test and p<0.05 was considered significant.

## Supporting Information

Table S1 **Slime production by the** ***S. epidermidis*** **strains used in this study on Congo-red agar.** Slime producing strains appear as dark black colonies. *S. epidermidis* ATCC 12228 was included as a negative control, not producing any slime and appeared as pink colonies.
(TIF)

Video S1 **Simultaneous growth of** ***S. aureus*** **ATCC 12600 and U2OS cells on PMMA surface for 48 h.**
(WMV)

Video S2 **Simultaneous growth of** ***S. epidermidis*** **ATCC 35983 and U2OS cells on PMMA surface for 48 h.**
(WMV)

Video S3 **Simultaneous growth of** ***S. aureus*** **ATCC 12600 and U2OS cells in the presence of macrophages on PMMA surface for 24 h.**
(WMV)
