# Supplementary figures and images for: In Vitro Interactions between Bacteria, Osteoblast-Like Cells and Macrophages in the Pathogenesis of Biomaterial-Associated Infections
Source: PLoS One. 2011 Sep 13;6(9):e24827. doi: 10.1371/journal.pone.0024827 (PMC3172284; doi:10.1371/journal.pone.0024827)

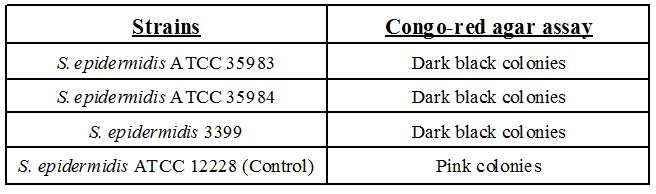

Supplement: Table S1 — Slime production by the S. epidermidis strains used in this study on Congo-red agar. Slime producing strains appear as dark black colonies. S. epidermidis ATCC 12228 was included as a negative control, not producing any slime and appeared as pink colonies. (TIF) [file pone.0024827.s001.tif]
